# Continental Survey of Access to Diagnostic Tools and Endovascular Management of Aneurysmal Subarachnoid Hemorrhage in Africa

**DOI:** 10.3389/fsurg.2021.690714

**Published:** 2021-07-20

**Authors:** Yao Christian Hugues Dokponou, Jay Kotecha, Soham Bandyopadhyay, Joshua Erhabor, Setthasorn Zhi Yang Ooi, Abdullah Egiz, Mahjouba Boutarbouch, David Ulrich Dalle, George Higginbotham, Mbaye Thioub, Dawin Sichimba, Nourou Dine Adeniran Bankole, Ulrick Sidney Kanmounye

**Affiliations:** ^1^Research Department, Association of Future African Neurosurgeons, Yaounde, Cameroon; ^2^Department of Neurosurgery, Mohammed V University, Rabat, Morocco

**Keywords:** aneurysmal subarachnoid hemorrhage, diagnostic tools, management, survey, Africa

## Abstract

**Rationale:** Interventional neurovascular procedures are effective in lowering the burden of mortality and complications resulting from aneurysmal subarachnoid hemorrhage (aSAH). Despite the wide uptake of interventional neurovascular procedures in high-income countries, access to care in low- and middle-income countries remains limited, and little is known about accessibility in Africa. In this survey, we decided to assess access to diagnostic tools and treatment of aSAH in Africa.

**Methodology:** A Google form e-survey was distributed to African neurosurgery centers accepting responses from January 4th to March 21st 2021. Data on accessibility to diagnostic tools, treatment methodologies, and interventional neuroradiology personnel in African centers were collected. Ninety five percent confidence intervals were computed for each variable.

**Results:** Data was received from 36 neurosurgical centers in 16 African countries (16/54, 30%). Most centers were public institutions. Ninety four percent of the centers had the necessary resources for a lumbar puncture (LP) and a laboratory for the diagnosis of aSAH. Most centers had at least one computed tomography (CT) scanner, 81% of the centers had access to CT angiography and some had access to conventional angiography. Forty seven percent of the centers could obtain a head CT within 2 h of presentation in an emergency. Sixty one percent of centers provided clipping of intracranial aneurysms whilst only 22% of centers could perform the endovascular treatment. Sixty four percent of centers did not have an endovascular specialist.

**Conclusion:** This survey highlights health inequity in access to endovascular treatment for aSAH. Lack of diagnostic tools to identify an aneurysm and a shortfall of qualified endovascular specialists are prime reasons for this. Our findings can inform health system strengthening policies including the acquisition of equipment and capacity building in Africa.

## Introduction

Subarachnoid hemorrhage (SAH) accounts for up to 8.9% of the total global stroke burden (1). Although SAH typically results from traumatic brain injury, it can also present subsequent to spontaneous rupture of an intracranial aneurysm where it is termed aneurysmal subarachnoid hemorrhage (aSAH). Timely access to computed tomography (CT), CT angiography (CTA), lumbar puncture, and/or conventional angiography is required to make a diagnosis of aSAH. Also, timely access to treatment is essential. Endovascular procedures have become the preferred treatment for most areas due to their less invasive nature, the reduced risk of postoperative complications, and favorable outcomes when compared to clipping (2).

Mortality resulting from aSAH remains high across Africa in comparison to high-income countries. The 1-month mortality rate from aSAH in Kenya is reported to be 26.6% whilst it is 44.4% in Nigeria with an incidence of 4.1 per 100,000 person-years. (3–5). Autopsy studies in Africa revealed up to 15.7% of cardiovascular causes of death in Kenya could be as a result of SAH, highlighting the large number of undiagnosed SAH cases in Africa (6). Hence the true incidence of SAH and aSAH is unknown and this may be due to delayed presentation and lack of access to neuroimaging and angiography as well as poor medical record keeping (4). In comparison, high-income countries (HICs) report in-hospital mortality rates ranging from 11.3 to 18% (7, 8). However, it should be noted that HICs report a 6 month mortality rate that is considerably higher than the in-hospital mortality rate and many more patients have unfavorable outcomes at follow-up (7, 8). Many HIC neurosurgical centers are able to provide timely access to neurosurgical treatment for aSAH, however; it is unclear if the same is true for neurosurgical centers across the African continent (9). It is reported that neurosurgical centers specializing in the treatment of intracranial aneurysms and aSAH are scarce in some African countries, and those that do exist only offer open vascular aSAH treatment (10). Indeed some African centers report needing to transfer patients to departments that can provide treatment for aSAH and this is particularly true for endovascular treatment (3, 11). This can sometimes result in patients being transferred to other countries or even another continent for appropriate treatment; albeit the literature does not mention the mode of transportation (12). Due to these factors and the delay in presentation, aSAH patients experience limited access to and significant delays in receiving endovascular treatment but also surgical clipping, and this is particularly true for public hospitals compared to private hospitals (4). The higher cost of endovascular procedures compared to clipping may impact the ability of low- and middle-income countries (LMICs) to provide such treatment (10, 13). Scarcity of safe and suitable medical equipment is another commonly cited barrier to the provision of endovascular treatment options (10). Furthermore, the lack of interventional neuroradiology (INR) training across Africa has resulted in a deficit of INR specialists across the continent (14). In fact, it is reported that patients with aSAH are often treated medically instead of procedurally in some African countries (15).

The tools required to diagnose aSAH such as neuroimaging, lumbar puncture, and angiography may be accessible in some African countries (16, 17). However, accessibility and availability of CT and MRI is highly variable across the African continent and can be significantly short of the recommended standard set by the World Health Organization (WHO) (18, 19). Neuroimaging is particularly scarce in rural African hospitals (20, 21). It is promising that some African hospitals do have access to CTA, though its availability remains limited and the cost of CTA remains unaffordable to many patients (22). aSAH mandates timely diagnosis in order for adequate treatment to be provided and to reduce the risk of further complications. As a result, African neurosurgical centers must have prompt access to diagnostic tools for aSAH.

The availability of endovascular treatment options and equally diagnostic tools for aSAH across Africa is unknown. There is a paucity of data as many African countries do not have published literature relating to these topics (15). It is hypothesized that many African countries face challenges in providing timely diagnosis and suitable treatment of aSAH. Knowledge of the presently available treatment modalities and diagnostic tools across Africa would allow for efficient resource allocation, workforce planning and to identify those centers that are in particular need of neurosurgical equipment donation. We aimed to address this knowledge gap and to create a map of the availability of endovascular treatments and diagnostic tools for aSAH across the African continent.

## Methods

A Google form e-survey questionnaire was distributed to African neurosurgical centers *via* social media platforms (WhatsApp, Telegram, LinkedIn, and Twitter). The survey was validated independently by two African professors of neurosurgery. The participants were identified through neurosurgical societies such as Association of Future African Neurosurgeons (AFAN) and Young African Neurosurgeons Forum (Young CAANS). The data collected included the submitter's role within the center, details about the healthcare setting, the methods of diagnosis, treatment of aSAH used locally, and the accessibility of said methods. Reminders to fill in the survey were sent every week. The survey was active from January 4, 2021 to March 21, 2021. Forty-nine responses were received in total. Thirteen responses (27%) were duplicate responses from the same centers. Duplicate data was removed via a standardized process by considering the seniority of the respondent, time in current role and the amount of data fields completed in the survey. Results were analyzed by calculating percentage data and by calculation of the mean, median and interquartile range where appropriate.

Ethics approval for this study was not needed as no personal information was being collected, but objective information about institutions.

## Results

**Figure 1:**
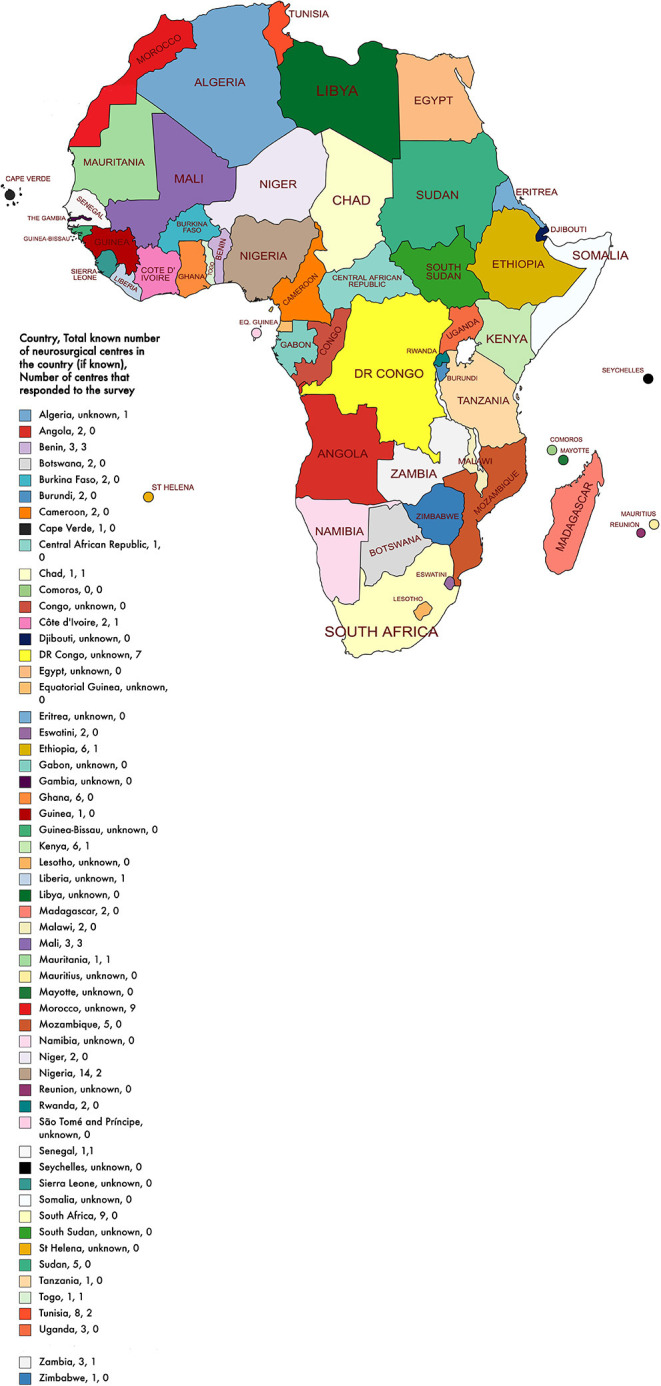
African map with country, total known number of neurosurgical centers in the country, and number of centers that responded to the survey.

After the removal of the duplicate responses, we received 36 responses. Data were received from 36 different neurosurgical centers in 16 of the 54 African countries (16/54, 30%) (Figure 1). Most institutions were public healthcare centers (24/36, 67%) whilst the remaining were either a private, military, or a mixed (public and private) center (Table 1). The median number of total beds at a center was 385 beds and the median number of intensive care unit beds at a center was 14 beds.

**Table 1:** Location (country) of surveyed neurosurgical centers, type of hospital, the total number of hospital beds at surveyed neurosurgical centers, total number of intensive care beds at surveyed neurosurgical centers.

**Country**	**Percentage of centers (actual)**
Algeria Benin Chad Côte d'Ivoire Democratic republic of the Congo Ethiopia Kenya Liberia Mali Mauritania Morocco Nigeria Senegal Togo Tunisia Zambia	3% (1) 8% (3) 3% (1) 3% (1) 19% (7) 3% (1) 3% (1) 3% (1) 8% (3) 3% (1) 25% (9) 6% (2) 3% (1) 3% (1) 6% (2) 3% (1)
**Type of hospital**	**Percentage of centers (actual)**
Public Private Both public/private Military Unknown	67% (24) 8% (3) 19% (7) 3% (1) 3% (1)
**Total number of hospital beds (range)**	**Percentage of centers (actual)**
1–50 51–200 201–400 401–700 701–1000 1,001+ Unknown	28% (10) 11% (4) 17% (6) 17% (6) 11% (4) 3% (1) 14% (5)
**Total number of ICU/ITU beds (range)**	**Percentage of centers (actual)**
1–5 6–10 11–20 21–30 31–40 41+ Unknown	19% (7) 33% (12) 8% (2) 14% (5) 6% (2) 3% (1) 19% (7)

The majority of centers had easy access to resources necessary for a lumbar puncture (LP) and an on-site laboratory for the diagnosis of aSAH (34/36, 94%). Most centers (30/36, 83%) had at least one CT scanner.

Most centers investigated aSAH using computed tomography (CT) angiography (29/36, 81%) and some had access to conventional angiography (17/36, 47%) of centers had access to formal/traditional angiography. Reasons commonly cited for lack of access to formal/traditional angiography were lack of equipment and lack of trained personnel (Table 2). Almost every center had access to a CT scan in their radiology department or at an external site in their city (35/36, 97%). Some of the centers could obtain a head CT within 2 h (17/36, 47%). A minority of centers lacked a CT scanner (6/36, 17%) in their radiology department, but all had access to a CT scanner in their city. Two of these centers (2/6, 33%) were able to perform an emergency CT head scan in 2–6 h, another two centers (2/6, 33%) could get a scan in 6–12 h, one (1/6) could do so in 12–24 h and the last center (1/6) needed more than 24 h to get a scan (Table 3). The majority of centers had access to magnetic resonance imaging (MRI) (33/36, 92%), whilst 67% (24/36) of centers had access to magnetic resonance angiography (MRA).

**Table 2:** Comments provided by respondents for reasons why formal/traditional cerebral angiography was not available at their center.

**Comments provided by respondents**
Unavailable in my hospital
Equipment and expertise not available
Technical platform not available
No available technical materials or human resources
Lack of resources by the government
Not available in our hospital so we refer the patient to a private clinic or another hospital for investigation
Not trained

**Table 3:** Clinical parameters and investigations that are typically used by the surveyed neurosurgical centers to diagnose subarachnoid hemorrhage (SAH), typical investigations utilized to investigate for an aneurysm in patients with SAH at the surveyed neurosurgical centers, time is taken to perform a CT head scan in an emergency, number of CT scanners at the surveyed neurosurgical centers, number of qualified interventional neuroradiology (INR) doctors at the surveyed neurosurgical centers.

**Typical investigations used to diagnose SAH**	**Percentage of centers (actual)**
Clinical evidence CT head CT intracranial angiogram Lumbar puncture	72% (26) 97% (35) 39% (14) 28% (10)
**Typical investigations used to investigate for an intracranial aneurysm following diagnosis of SAH**	**Percentage of centers (actual)**
CT intracranial angiogram Conventional cerebral angiography No available imaging modality	81% (29) 25% (9) 17% (6)
**Time to CT head in an emergency**	**Percentage of centers (actual)**
<1 h 1–2 h 2–6 h 6–12 h 12–24 h 24+ h 1 week 1 month	3% (1) 44% (16) 22% (8) 17% (6) 6% (2) 8% (3) 0% (0) 0% (0)
**Number of CT scanners**	**Percentage of centers (actual)**
0 1 2 3 4+	17% (6) 50% (18) 25% (9) 8% (3) 0% (0)
**Number of qualified INR doctors**	**Percentage of centers (actual)**
0 1 2 3 4+	64% (23) 8% (3) 8% (3) 11% (4) 8% (3)

Twenty-two centers performed clipping (22/36, 61%) whereas only 22% (8/36) of centers offered endovascular treatment. Almost two-thirds of centers did not have an endovascular specialist in their center (23/36, 64%). Centers that provided endovascular treatment were teaching or university-affiliated hospitals. All of these centers had at least 1 on-site CT scanner, access to MRI, MRA, and formal angiography. Six of these centers reported taking a maximum of 2 h to obtain a CT head whilst the remaining 2 centers reported taking 2–6 h and 6–12 h, respectively. The number of beds at these centers ranges from 14 to 2,535 (median = 240, IQR = 600–29) with the number of ITU beds ranging from 4 to 20 (median = 8, IQR = 22.5–5.5).

Of the 22 centers providing open vascular aneurysmal treatment, 45% (10/22) provided service on weekends and 41% (9/22) provided service out of regular working hours. Of the eight centers providing endovascular treatment for aSAH, 63% (5/8) performed endovascular treatment on weekends and out of regular working hours.

## Discussion

To our knowledge, this is the first continental survey to inventory the diagnostic tools and endovascular treatment of aSAH across Africa. We found that almost every neurosurgical center had access to a CT scan in their radiology department or at an external site in their city and nearly half of the centers diagnosed SAH via a CT scan within 1–2 h of presentation. Clipping was the more common method of securing intracranial aneurysms. Notably, only 22% of centers could provide endovascular treatment. The majority of centers did not have an endovascular specialist at their center. Overall the duplicate responses received from the neurosurgical centers were very similar in their responses though neurosurgical trainees tended to be slightly more optimistic than a consultant/attending neurosurgeon from the same center when considering the time taken to obtain a CT head in an emergency. Our study highlights the lack of endovascular treatment options available to treat aSAH across the African continent.

The disparity of neurosurgical outcomes can be a result of a lack of accessibility to the innovative neuroimaging modalities which aid operative accuracy and postoperative outcomes (23). Our continental survey reported similar findings to Sader et al. (24) as the authors reported 95 and 81% of neurosurgical centers in Sub-Saharan Africa had access to CT scanners and MRI, respectively, compared to 97 and 92% in our survey, respectively. Furthermore, from the 33 (33/36, 92%) centers that can carry out MRI, 11 (11/33, 33%) centers reported not having access to magnetic resonance angiography (MRA) without providing the reason for this. It is known that there is a shortage of MRI scanners across Africa and the majority of MRI units have low-field scanners (25). Reasons for lack of access to MRA may include lack of MRA software and lack of radiologists trained in interpreting MRA images. Furthermore, some neurosurgical centers may transfer patients to an external MRI unit for imaging but these units may not be able to provide advanced imaging such as MRA. Current global guidelines recommend non-contrast CT head as the cornerstone for diagnosing aSAH with a sensitivity of almost 100% within the first 3 days (26). This matches the majority of our survey responses as the timing of the emergency CT head was reported to be within 2 h of presentation in 47% (17/36) compared to 45% (16/36) within 2–24 h and 8% (3/36) in more than 24 h. However, it is important to consider travel times to neurosurgical centers since the time of ictus, as longer travel times are associated with poorer outcomes (27).

Around 10 countries did not have more than one neurosurgical center (28–30). Only 22% of the neurosurgical centers were able to perform the endovascular procedures recommended for aSAH. Thus, the majority of the patients will experience time delays in the form of (a) waiting for an ambulance to be transferred to a neurointerventional center; (b) long travel distance to a neurointerventional center; (c) reassessment by the neurointerventional center's team. Although our continental survey did not aim to provide a comparative analysis of post-procedure outcomes between the endovascular treatment and surgical intervention for aSAH, the endovascular approach is less invasive in nature and has reduced risk of postoperative complications compared to clipping (2).

Additional factors may play a vital role in increasing the time from diagnosis to treatment. Germans et al. conducted a multivariate analysis showing that presentation at a referring hospital and admission to the treatment center later in the day were independently related to a longer time interval between diagnosis and treatment (31). Our survey showed that 45% of neurosurgery centers provided service on weekends and 41% provided service out of regular working hours. Moreover, only five (13.9%) of the 36 surveyed units provided endovascular treatment on the weekends. Therefore, the majority of the patients admitted with aSAH across Africa over the weekend are at high risk of experiencing treatment delays. Patients with severe aSAH (poor neurological grade) who are left untreated over a weekend are associated with an independent risk of mortality within 12 weeks of the onset of symptoms (32). Interestingly of the five centers providing weekend endovascular treatment, four (80%) were based in Morocco; this was 44.4% (4/9) of the neurosurgical units that we surveyed in Morocco. The disparity in the accessibility and availability of endovascular services within Africa further compounds the issue for patients residing outside Morocco.

The underlying factors to the shortage of endovascular services could be due to reasons such as the cost of materials, lack of INR specialists, inadequate INR training, and insufficient infrastructure. It is known that the cost of endovascular coiling is higher than clipping in LMICs, largely due to the cost of the materials needed for coiling (33). As a result, patients in LMICs may not be able to afford endovascular treatment. Furthermore, our study revealed that almost two-thirds of neurosurgery centers did not have an endovascular specialist at their center. Thus, an increased number of qualified INRs is needed to reduce the time to treatment. As INR is a newly developing field in neurosurgery requiring highly specialized training and access to advanced technology, it will be beneficial for Africa to further develop capacity-building efforts, gain training mentorship from global neurosurgeons and obtain funding support from global health actors and policymakers to promote upskilling of African neurosurgeons. Given the prevalence and the high mortality rate of aSAH in Africa, it is imperative that the growing neurosurgical workforce is equipped with the skills they need to meet the unmet burden of the disease. Compared to HICs, a lack of equipment and trained personnel in LMICs puts many neurosurgeons and neurosurgery centers at a disadvantage when it comes to efficiently treat aSAH using endovascular procedures. A lack of endovascular treatment options may also hinder the performance of neurosurgeons, increasing risk of burnout due to working more than their expected capacity. These barriers should be prioritized in future guidelines to address the inequalities of the healthcare standards provided across the continent.

The lack of INR specialists is not a problem that is limited to Africa and LMICs. HICs such as the United Kingdom (UK) face similar problems. Unlike many countries which produce hybrid neurosurgeons that perform both endovascular and open neurosurgical techniques, INR in the UK is rather considered as a subspecialty for those training in clinical radiology (34). It has been reported that there were only 90 trained INRs working in 28 neuroscience centers in the UK in 2017; this sparsity in the workforce may not provide comprehensive endovascular service nationally (35). This, too, has led to a shortfall in the delivery of endovascular treatment on weekends compared to weekdays, thus consequently leading to significantly longer mean waiting times from admission or scan to treatment time (both *p* < 0.0001) and higher in-hospital mortality rates (*p* = 0.08) (36). Interestingly, Kotecha et al. surveyed the attitudes of neurosurgical trainees in pursuing INR training and found that trainees have the interest and the insight to develop skills and knowledge in INR. Therefore, there is potential to improve the service provision of INR in HICs such as the UK, provided trainees are given the opportunity to pursue it (37). Future research could make use of a similar survey to assess the appetite of African neurosurgical trainees toward endovascular training. Such research could kindle a learning opportunity to develop between African neurosurgeons and global INR mentors, thereby upskilling the continental workforce.

We collected data from 16 African countries; less than half of the total number of African countries. Major neurosurgical hubs like, Egypt, South Africa, Tunisia, and Zimbabwe were not represented in this study. The precise locations and the number of all active neurosurgical units across Africa is not known and so we were unable to calculate the proportion of African neurosurgical centers that responded to the survey. However, data on the spread of neurosurgeons across Africa exists and we received responses from the majority of African countries with more than four practicing neurosurgeons (38). Furthermore, 1 in 4 of our responses was from Morocco (25%, *n* = 9). This could have skewed the results as Morocco had better access to endovascular treatment services compared to the rest of the countries surveyed. Moreover, the small sample size means the results of the study are not entirely representative of the African continent. The small sample size equally limited our ability to compute disaggregated data and we could not calculate comparative differences at the regional level. To minimize the effect of non-response bias we increased the data collection period and the number of survey dissemination modes.

## Conclusion

The diagnosis and treatment of aSAH are well-established. The findings of this study demonstrate a lack of availability and accessibility of diagnostic and management tools for aSAH in the surveyed African neurosurgical centers. This is a detrimental factor not only to the health of Africans requiring intervention but also to the World Health Organisation's (WHO) initiative in achieving universal health coverage. This study highlights the disparity of endovascular care uptake in comparison to high-income countries as well as within African countries. This study also finds that neurosurgical clipping is unavailable in many African neurosurgical centers. We urge local governments and stakeholders to review their specialty training curricula and to invest in expanding the infrastructure of the neurosurgical units in their countries, to ensure that all patients with aSAH can access quality and timely intervention, regardless of their location or time of bleed. Initially this can be achieved by expanding the availability of neurosurgical clipping across Africa whilst an endovascular treatment infrastructure is developed.

## Data Availability Statement

The datasets presented in this study can be found in online repositories. The names of the repository/repositories and accession number(s) can be found in the article/supplementary material.

## Author Contributions

YD: Conceptualization, Data curation, Writing the original draft, Reviewing, editing, and Project administration. JK: Conceptualization, Writing the original draft, Data analysis, Reviewing, editing, and Project administration. AE, SO, and JE: Writing the original draft. SB: Conceptualization, Reviewing, editing, and Supervision. DD and DS: Visualisation. MB and MT: Visualisation and Validation. GH: Conceptualisation. NB: Conceptualisation, Data curation, and Supervision. UK: Conceptualisation, Data analysis, Writing draft, Visualisation, and Supervision. All authors contributed to the article and approved the submitted version.

## Conflict of Interest

The authors declare that the research was conducted in the absence of any commercial or financial relationships that could be construed as a potential conflict of interest.
